# Hsa_circ_0068307 mediates bladder cancer stem cell-like properties via miR-147/c-Myc axis regulation

**DOI:** 10.1186/s12935-020-01235-6

**Published:** 2020-05-06

**Authors:** Qi Chen, Qiuping Yin, Yemeng Mao, Zheyu Zhang, Siqi Wu, Zhang Cheng, Xinan Chen, Hanren Xu, Shengming Jin, Haowen Jiang, Chen Yang

**Affiliations:** 1 Department of Urology, Huashan Hospital, Fudan University, Shanghai, China; 2 Fudan Institute of Urology, Huashan Hospital, Fudan University, Shanghai, China; 3 Shanghai Mental Health Center, Shanghai Jiaotong University School of Medicine, Shanghai, China; 4 Department of Urology, Zhongshan Hospital, Fudan University, Shanghai, China; 5 Shanghai Cancer Center, Fudan University, Shanghai, China; 6 National Clinical Research Center for Aging and Medicine, Fudan University, Shanghai, China

**Keywords:** Hsa_circ_0068307, Bladder cancer, miR-147, c-Myc, Cancer stem cell

## Abstract

**Background:**

Circular RNAs (circRNAs) play an essential role in the regulation of gene expression. However, the underlying mechanisms remain unknown. This study aimed to evaluate the role of hsa_circ_0068307 in bladder cancer (BCa).

**Methods:**

Rt-qPCR was used to detect hsa_circ_0068307 expression in BCa cell lines. The CCK8, colony formation, and Transwell assays were used to evaluate the effect of hsa_circ_0068307 on BCa cell migration and proliferation. Bioinformatics and luciferase reporter experiments were used to study the regulatory mechanism. Nude mouse xenografts were generated to examine the effect of hsa_circ_0068307 on tumor growth.

**Results:**

The results showed that hsa_circ_0068307 was upregulated in BCa cell lines. Downregulation of hsa_circ_0068307 suppressed cell migration and proliferation in T24 and UMUC3 cells. Hsa_circ_0068307 silencing suppressed cancer stem cell differentiation by upregulating miR-147 expression. Upregulation of miR-147 suppressed c-Myc expression, which is involved in cancer stem cell differentiation. Luciferase reporter assays confirmed that hsa_circ_0068307 upregulated c-Myc expression by targeting miR-147. In vivo studies showed that hsa_circ_0068307 knockdown suppressed T24 tumor growth.

**Conclusions:**

These data indicate that downregulation of hsa_circ_0068307 reversed the stem cell-like properties of human bladder cancer through the regulation of the miR-147/c-Myc axis.

## Background

Bladder cancer (BCa) is a common malignancy worldwide, with 430,000 new cases diagnosed in 2012 [1]. In China, BCa was the sixth most common cancer among men in 2015 [2]. Approximately 75% of newly diagnosed BCas are non-muscle invasive, and 70% of them experience relapse, with 25% progressing to muscle-invasive BCa [1]. Because of the high incidence, progression, and recurrence rates, the 5-year survival rate of BCa patients is low [3]. Therefore, elucidating the mechanisms that contribute to metastasis, progression, and development of BCa is indispensable.

Circular RNAs (circRNAs) are a newly discovered of endogenous noncoding RNAs (ncRNAs) constructed from introns or exons via particular selective shearing. Derived from linear RNA, circRNAs consist of covalently closed loop structures that have unique, highly stable, and diverse forms [4–6]. circRNAs have critical functions in many pathological and biological processes, including cell migration, invasion, and proliferation, cell cycle progression, metastasis, and carcinogenesis [7–10]. Previous studies showed that circCASC15 may function as a miR-1224-5p sponge to activate CREB1 expression and promote cell proliferation in BCa [11]. CircHIPK3 sponges miR-558 to suppress BCa cell heparanase expression [12]. However, the role of hsa_circ_0068307 in BCa is unknown.

Cancer stem cells (CSCs) are important for tumor initiation and development [13]. CSCs express specific markers such as Oct4, Sox2, and NANOG [14]. Targeting CSCs is a promising and novel strategy for the treatment of tumors that are resistant to known therapies, which lead to treatment failure or recurrence [15]. Circ008913 participates in CSC-like property acquisition induced by arsenite through keratinocytes, thereby affecting carcinogenesis via miR-889 DAB2IP/ZEB1 regulation [16]. However, the role of hsa_circ_0068307 in the regulation of BCa development by CSCs remains unknown. The purpose of this study was to elucidate the hsa_circ_0068307 regulatory mechanism using in vitro and in vivo experiments.

## Materials and methods

### Animal ethics statement

Four-week-old nude BALB/c mice (n = 12) weighing 15–20 g (SLARC, Shanghai, China) were used. The Ethics Committee of Huashan Hospital, Fudan University approved all animal experiments.

### Tissue samples

In total, 30 fresh Bca and paired adjacent noncancerous BCa tissues were collected after obtaining informed consent from patients at Huashan Hospital, Fudan University. Samples were snap-frozen in liquid nitrogen and stored at − 80 °C before RNA extraction. Huashan Hospital Ethics Committee in Fudan University approved this study.

### Cell culture and transfection

BC and SV-HUC cell lines (EJ, T24, RT-4 and UM-UC-3) were obtained from the Type Culture Collection of the Chinese Academy of Sciences, Shanghai and cultured at 37 °C in 5% CO_2_ in DMEM medium (Gibco, Gaithersburg, MD, USA) containing 10% fetal bovine serum (FBS; Gibco). T24 cells were transfected with miR-147 mimics, small interfering RNAs (siRNAs), miR-147 inhibitors, c-Myc overexpression vector, and negative controls using Lipofectamine 2000 (Invitrogen, Carlsbad, CA, USA), according to the standard procedure. Full length fragment of c-Myc was constructed into a GV358 vector to induce c-Myc overexpression (Genechem, Shanghai, China). To confirm the hsa_circ_0068307 effect in in vivo experiments, we constructed lentiviral stabilized hsa_circ_0068307 silenced T24 cells through PAX2/PMD2G packing vectors, while shRNA targeting hsa_circ_0068307 was cloned into pLKD-CMV-EGFP vectors (GeneWiz, Guangzhou, China).

- si-NC sense: UUCUCCGAACGUGUCACGUTT.
- si-NC antisense: ACGUGACACGUUCGGAGAATT.
- si-circ sense: CUCAAGCAGUACACAGAGATT.
- si-circ antisens: UCUCUGUGUACUGCUUGTGTT.

### Migration assay

Transwell chamber migration assays were performed. In brief, cells were resuspended with culture medium including 1% FBS (Biological Industries, Cromwell, CT, USA), and 200 µL cell suspension (1 × 10^5^ cells) was added into the Transwell chamber (BD Biosciences, Franklin Lakes, NJ, USA). The lower chamber was filled with 500 µL medium containing FBS at 30%. After incubation for 1 day at 37 °C with 5% CO_2_, cells were rinsed with PBS and fixed in 0.5% methanol for 30 min, then stained with crystal violet 0.1% for 20 min, and rinsed with PBS. Non-invading cells were removed using a cotton swab, and migrating cells were observed by microscopy and counted. All experiments were performed in triplicate.

### Bioinformatics analysis

We predicted circRNA/miRNA target genes via the online tool, *Circular RNA Interactome*. We predicted interacting relationships between miR-147 and c-Myc through the web-based package, *TargetScanHuman*.

### Cloning formation assay and cell proliferation assay

The Cell Counting Kit-8 (CCK-8) assay was used to explore cell proliferation. Transfected cells were seeded into triplicate wells of 96-well plates at a density of 2000 cells/well. We measured cell viability via the CCK-8 system (Gibco) at 0, 24, 48, 72, and 96 h after seeding, following the standard procedure.

Transfected cells were seeded into six-well plates at a density of 2000 cells per well in DMEM containing FBS at 10% for 10 days for the colony formation assay. Colonies were imaged and counted after fixing and staining.

### Western blots

Protein was extracted from cells and tumor tissues using RIPA lysis buffer containing protease inhibitors (Sigma-Aldrich, St. Louis, MO, USA) and the BCA Protein Assay kit (Vigorous Biotechnology Beijing, Beijing, China) was used to measure protein concentration. Twenty micrograms of protein per sample were separated using SDS-PAGE and transferred onto nitrocellulose membranes (Millipore, Madison, WI, USA), which were blocked with 5% nonfat dry milk for 2 h before incubation with primary antibodies at 4 °C overnight. Glyceraldehyde 3-phosphate dehydrogenase was used as the internal control. Membranes were incubated with horseradish peroxidase-conjugated secondary antibodies for 1 h at room temperature.

### RNA extraction and quantitative reverse transcription-polymerase chain reaction (RT-qPCR)

RNA extraction was performed using the TRIzol reagent (Invitrogen), as described in Li et al. [17]. cDNA was obtained using the pTRUEscript 1st Strand cDNA Synthesis Kit (Aidlab, Beijing, China). qRt-PCR was performed using the 2× SYBR Green qPCR Mix (Aidlab) on an ABI 7900HT sequence detection instrument (Thermo Fisher Scientific, Waltham, MA, USA). Fold-changes in expression were calculated using the 2^−ΔΔCT^ method.

### Luciferase reporter assay

Wild type (WT) hsa_circ_0068307 cDNA fragments, including the predicted miR-147 binding sites were amplified and mutated fragments (Mutant) were generated by overlap extension PCR. WT and mutant fragments were cloned into the psiCHECK-2 (Promega, Madison, WI, USA). HEK293T cells were co-transfected with WT or mutant vector and miR-147 or control mimics using Lipofectamine 2000 (Thermo Fisher Scientific) for luciferase reporter assays. After 2 days of transfection, luciferase activity was detected using a dual-luciferase reporter assay kit (Promega) and normalized to Renilla luciferase activity. All experiments were performed in triplicate.

### Metastasis assays and tumor xenograft formation

In total, we injected 2 × 10^7^ viable cells from wild-type or sh-circRNA T24 cells into the right flanks of 12 nude mice [18]. We measured tumor sizes every 5 days using a Vernier caliper. Tumor volume was calculated using the following formula: volume = 0.5 * width^2^ * Length. Mice were euthanized for qRT-PCR analyses 1 month after implantation.

### Tumor sphere formation assay

Cells were harvested and re-suspended as single cells in a non-serum medium (as previously mention in Tumor specimens and cell culture). After accurate cell counting, 200 cells/well in 200 μL of non-serum medium were added to a 96 well plate, and each group was in 10 wells. The medium was change every 2 days. Image of five randomly selected regions of each group were taken with a microplate reader (Leica, Wetzlar, Germany). The sphere percentage was calculated as the number of sphere/200.

### Statistical analysis

Data were expressed as the mean ± standard deviation (SD). GraphPad Prism (version 5.0; GraphPad, La Jolla, CA, USA) was used to compare differences among groups. A *P* value ≤ 0.05 inferred statistical significance.

## Results

### Hsa_circ_0068307 is highly expressed in BCa and exerts oncogenic effects in UMUC3 and T24 BCa cell lines

**Fig. 1:**
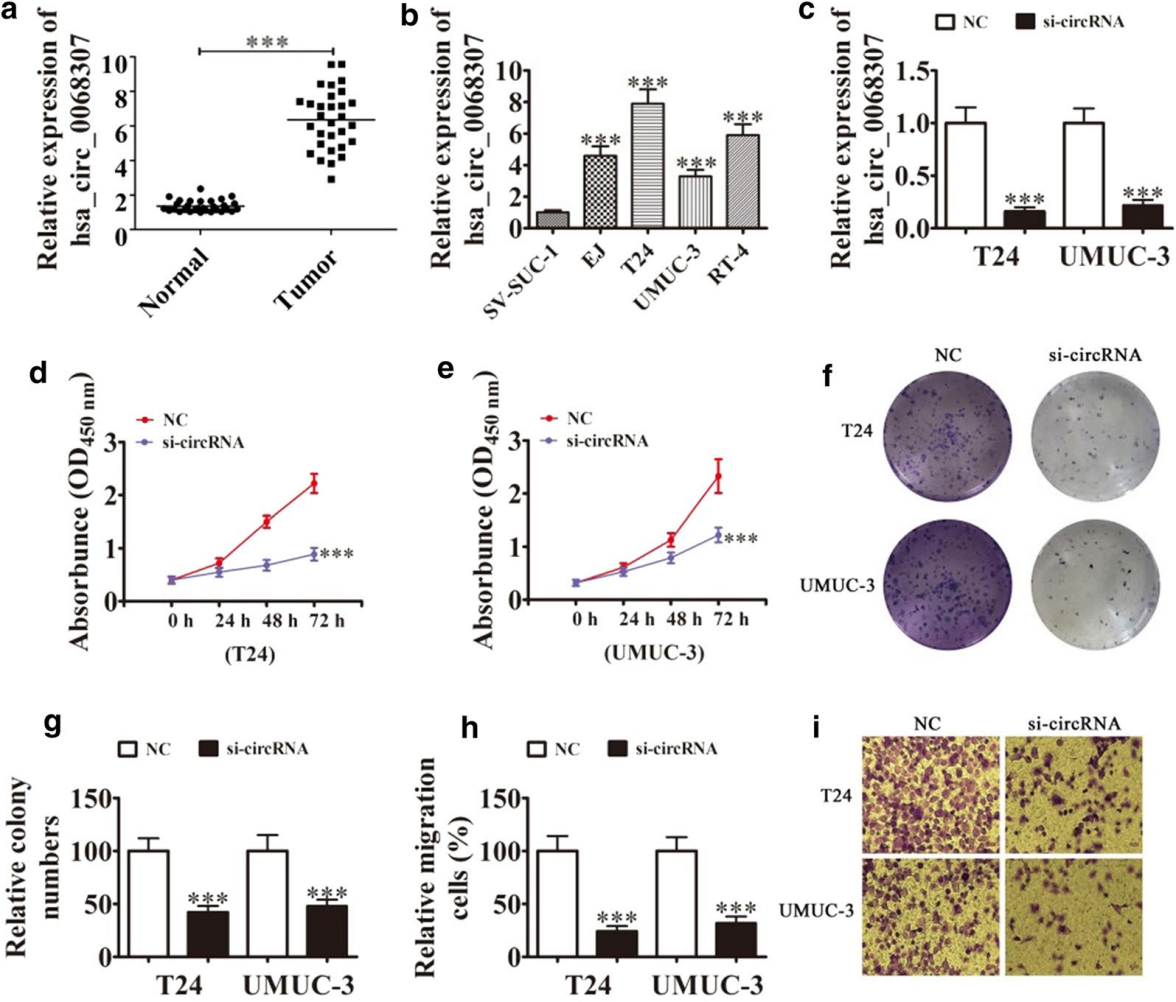
Hsa_circ_0068307 is expressed at high levels in BCa and exerts oncogenic effects in the BCa cell lines T24 and UMUC3. **a** Rt-qPCR detection showing the expression of hsa_circ_0068307 in tumor tissues and adjacent normal tissues. Data are presented as the mean ± SD. ***P < 0.001. **b** Rt-qPCR detection showing the expression of hsa_circ_0068307 in SV-HUC-1 and BCa cell lines. Data are presented as the mean ± SD. ***P < 0.001 vs. SV-HUC-1. **c** The expression of hsa_circ_0068307 was detected in T24 and UMUC-3 cells transfected with siRNA hsa_circ_0068307 (si-circRNA) or negative control (NC). Data are presented as the mean ± SD. ***P < 0.001 vs. NC. **d**, **e** CCK8 detection showing that hsa_circ_0068307 knockdown suppressed cell proliferation in T24 (**d**) and UMUC-3 (**e**) cells. Data are presented as the mean ± SD. ***P < 0.001 vs. NC. **f**, **g** Colony formation assays showing the proliferation of T24 and UMUC3 cells after knockdown of hsa_circ_0068307. Data are presented as the mean ± SD. ***P < 0.001 vs. NC. **h**, **i** Transwell assays showing the migration of BCa cells after knockdown of hsa_circ_0068307. Data are presented as the mean ± SD. ***P < 0.001 vs. NC

Rt-qPCR detection showed that hsa_circ_0068307 expression was higher in BCa tissues than in adjacent normal tissues in our cohort (Fig. 1a). The results also showed that hsa_circ_0068307 expression was higher in the BCa cell lines EJ, RT-4, T24, and UMUC-3 than in SV-HUC-1 cells (Fig. 1b). Because, T24 and UMUC-3 cell have more higher hsa_circ_0068307 expression, so we selected T24 and UMUC-3 cells for further study. Cells were treated with siRNA against hsa_circ_0068307 (si-circRNA), and the result showed that hsa_circ_0068307 expression decreased significantly in both T24 and UMUC-3 cells (Fig. 1c). CCK8 detection (Fig. 1d, e) and colony formation assays (Fig. 1f, g) showed that hsa_circ_0068307 knockdown suppressed T24 and UMUC-3 cell proliferation. Transwell assays showed that hsa_circ_0068307 silencing decreased the migration ability of T24 and UMUC-3 cells (Fig. 1h, i). These data suggested that hsa_circ_0068307 is generally up-regulated in BCa clinical samples and cell lines, thus possesses a potential oncogenensis role in the progression of BCa.

### Hsa_circ_0068307 functions as a miR-147 sponge, and c-Myc is a direct miR-147 target

**Fig. 2:**
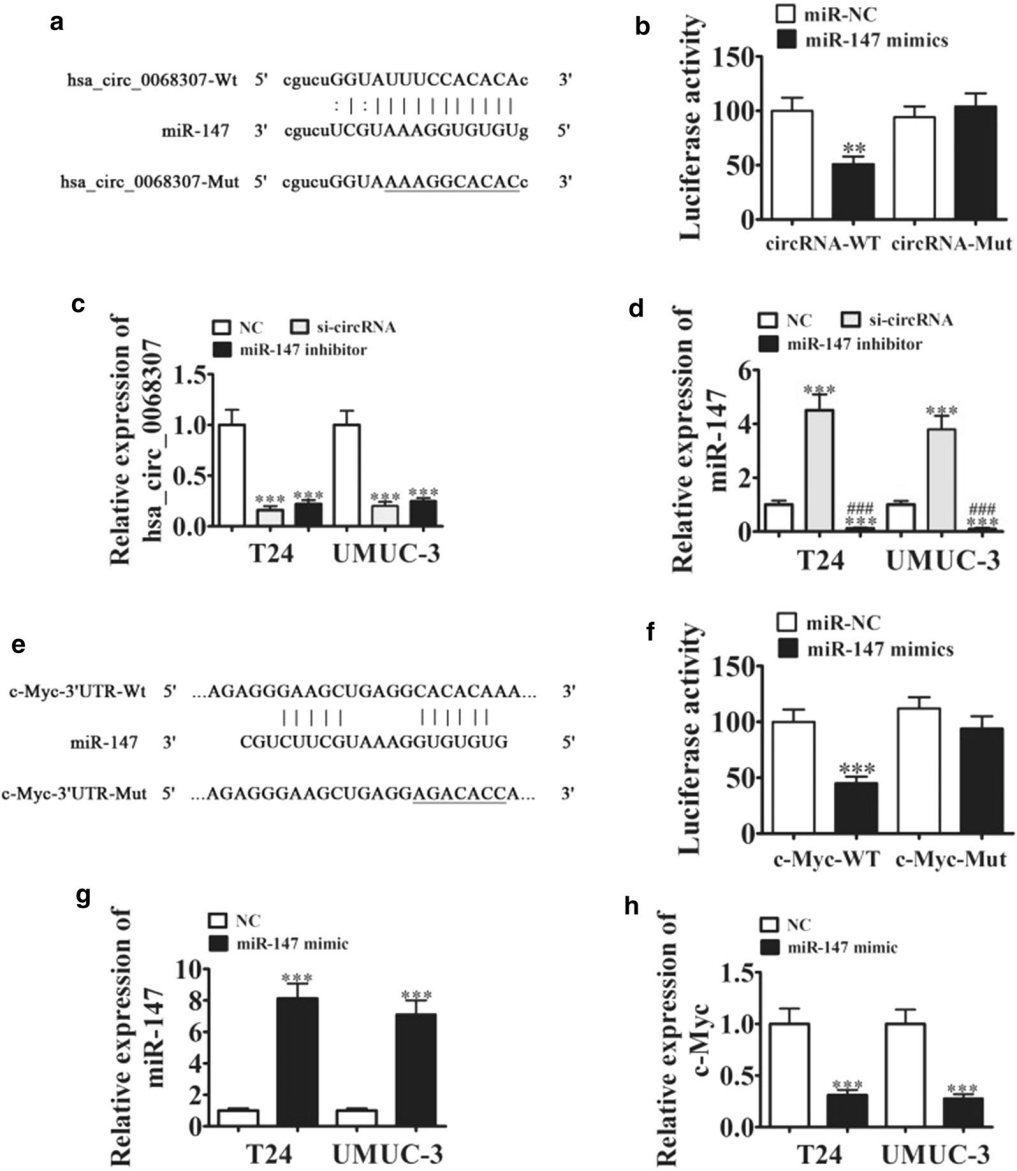
Hsa_circ_0068307 acts as a sponge for miR-147, and c-Myc is a direct target of miR-147. **a** The complementary sites within hsa_circ_0068307 and miR-147 were predicted by bioinformatics analysis. The mutated (Mut) version of hsa_circ_0068307 is also shown. **b** Dual luciferase reporter assays demonstrated that miR-147 is a direct target of hsa_circ_0068307. Data are presented as the mean ± SD. ***P < 0.001 vs. control. **c**, **d** qRt-PCR detection showing the expression of hsa_circ_0068307 and miR-147 in T24 and UMUC3 cells transfected with si-circRNA or miR-147 inhibitor. Data are presented as the mean ± SD. ***P < 0.001 vs. NC. ^###^P < 0.001 vs. si-circRNA. **e** The predicted binding sites of miR-147 with the 3′-UTR of c-Myc. The mutated version of the 3′-UTR-c-Myc is also shown. **f** Relative luciferase activity was determined 48 h after transfection with miR-147 mimic/normal control or with the 3′-UTR-c-Myc wild-type/Mut in HEK293T cells. Data are presented as the mean ± SD. ***P < 0.001 vs. control. **g**, **h** qRt-PCR detection showing the expression of miR-147 (**g**) and c-Myc (**h**) in T24 and UMUC3 cells transfected with miR-147 mimic. Data are presented as the mean ± SD. ***P < 0.001 vs. NC

Next, we explored the hsa_circ_0068307 regulatory mechanism involved in BCa progression. Bioinformatics analysis was used to predict the hsa_circ_0068307 targets, which showed an interacting relationship between hsa_circ_0068307 and miR-147. WT or mutated sequences containing the miR-147 binding sequence were used to construct a luciferase reporter vector (Fig. 2a). The luciferase reporter vector was transfected into 293T cells, combined with or without the miR-147 mimic. Luciferase reporter analysis showed that miR-147 inhibited the luciferase activity in WT cells, but not in mutated cell lines (Fig. 2b). This indicated that miR-147 was the target of hsa_circ_0068307. Rt-qPCR detection confirmed that hsa_circ_0068307 silencing suppressed hsa_circ_0068307 expression, and miR-147 inhibitor treatment failed to recover the expression of hsa_circ_0068307 (Fig. 2c). However, hsa_circ_0068307 silencing upregulated miR-147 expression in UMUC-3 and T24 cells. miR-147 inhibitor treatment suppressed the promotion effect of hsa_circ_0068307 silencing (Fig. 2d). These results suggested that miR-147 was the hsa_circ_0068307 downstream target.

Bioinformatics analysis identified a relationship between miR-147 and the 3′-UTR of c-Myc. A mutated or WT sequence containing the miR-147 binding site was used to construct a luciferase reporter vector (Fig. 2e). The luciferase reporter vector was transfected into 293T cells, combined with or without the miR-147 mimic. The results of luciferase reporter assays showed that miR-147 inhibited the luciferase activity in WT cells, but not in mutated cell lines (Fig. 2f), suggesting that miR-147 can interact with the 3′-UTR of c-Myc. Rt-qPCR detection confirmed that miR-147 overexpression (transfected with miR-147 mimics) upregulated miR-147 expression (Fig. 2g) and downregulated c-Myc (Fig. 2h). These results suggested that c-Myc was the downstream target of miR-147.

### Knockdown of hsa_circ_0068307 inhibits cancer stem cell-mediated BCa cell proliferation and migration by regulating the miR-147/c-Myc axis

**Fig. 3:**
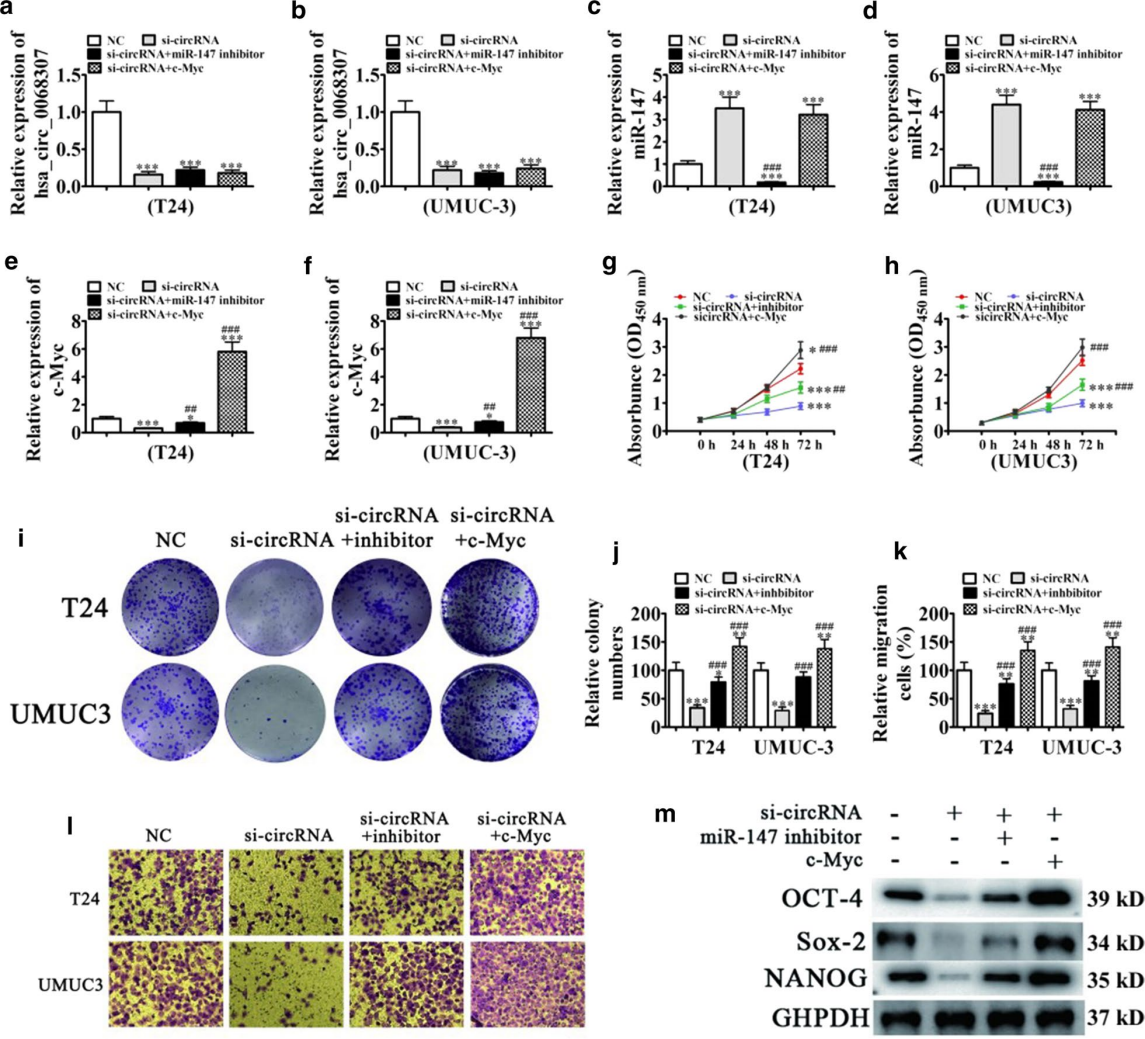
Knockdown of hsa_circ_0068307 inhibits cancer stem cell-mediated BCa cell proliferation and migration by regulating the miR-147/c-Myc axis in in vitro. **a**, **b** RT-qPCR assay showing hsa_circ_0068307 expression in T24 (**a**) and UMUC3 (**b**) cells after transfection with siRNA against hsa_circ_0000291, combined with or without miR-147 inhibitor or the c-Myc overexpression vector. Data are presented as the mean ± SD. ***P < 0.001 vs. NC. **c**, **d** RT-qPCR assay showing miR-147 expression in T24 (**c**) and UMUC3 (**d**) cells. Data are presented as the mean ± SD. ***P < 0.001 vs. NC. ^###^P < 0.001 vs. si-circRNA. **e**, **f** RT-qPCR detection showing c-Myc expression in T24 (**e**) and UMUC3 (**f**) cells. Relative protein levels were analyzed and data are presented as the mean ± SD. ***P < 0.001 vs. NC. ^###^P < 0.001 vs. si-circRNA. **g**, **h** CCK8 assays were performed to assess cell proliferation in T24 (**g**) and UMUC3 (**h**) cells. Data are presented as the mean ± SD. *P < 0.05, ***P < 0.001 vs. NC. ^###^P < 0.001 vs. si-circRNA. **i**, **j** Clone formation assay showing the proliferation of T24 and UMUC3 cells. Data are presented as the mean ± SD. **P < 0.01, ***P < 0.001 vs. NC. ^###^P < 0.001 vs. si-circRNA. **k**, **l** Cell migration was determined in T24 and UMUC3 cells using Transwell^®^ assays. Data are presented as the mean ± SD. **P < 0.01, ***P < 0.001 vs. NC. ^###^P < 0.001 vs. si-circRNA. **m** Western blow analysis showing the expression of cancer stem cell-related proteins OCT-4, Sox-2, and NANOG in T24 and UMUC3 cells. Relative protein levels were analyzed and data are presented as the mean ± SD. ***P < 0.001 vs. NC. ^###^P < 0.001 vs. si-circRNA

Previous work focus on the regulative role of c-Myc in cell self-renewal process and stemness maintenance [13]. Si-hsa_circ_0068307 T24 cells show relatively poorer sphere formation ability compared to si-NC T24 cells (Additional file 1: Figure S1), which indicates hsa_circ_0068307 correlates with cell stemness ability. We further evaluated whether the proliferation suppression effect of silencing hsa_circ_0068307 was mediated via miR-147/c-Myc axis through several rescue experiments. RT-qPCR assays showed that hsa_circ_0068307 silencing suppressed hsa_circ_0068307 and c-Myc expression and upregulated miR-147 expression. Combination treatment with the miR-147 inhibitor downregulated miR-147 expression and restored c-Myc expression. However, miR-147 inhibitor treatment had no effect on hsa_circ_0068307 expression. Transfection with c-Myc overexpression vector significantly increased c-Myc in both T24 and UMUC-3 cells, whereas it had no effect on hsa_circ_0068307 and miR-147 expression (Fig. 3a–f).

CCK8 detection (Fig. 3g, h) and colony formation assays (Fig. 3i, j) showed that miR-147 inhibitor treatment partially recovered hsa_circ_0068307 knockdown-induced decrease in proliferation ability. c-Myc overexpression increased the proliferation of both UMUC-3 and T24 cells even after hsa_circ_0068307 silencing. Transwell assays also showed that miR-147 inhibitor treatment partially recovered the hsa_circ_0068307 knockdown-induced decrease in migration ability. c-Myc overexpression promoted migration in UMUC-3 and T24 cells even after hsa_circ_0068307 silencing (Fig. 3k, l). Western blot analysis showed that miR-147 inhibitor treatment partially recovered the expression of the CSC-related proteins, NANOG, OCT-4, and Sox-2 after hsa_circ_0068307 silencing. c-Myc overexpression upregulated the expression of CSC-related proteins in UMUC-3 and T24 cells even after hsa_circ_0068307 silencing (Fig. 3m).

### c-Myc overexpression reverses the miR-147 mediated inhibition of cancer stem cell-mediated BCa cell migration and proliferation in vitro

**Fig. 4:**
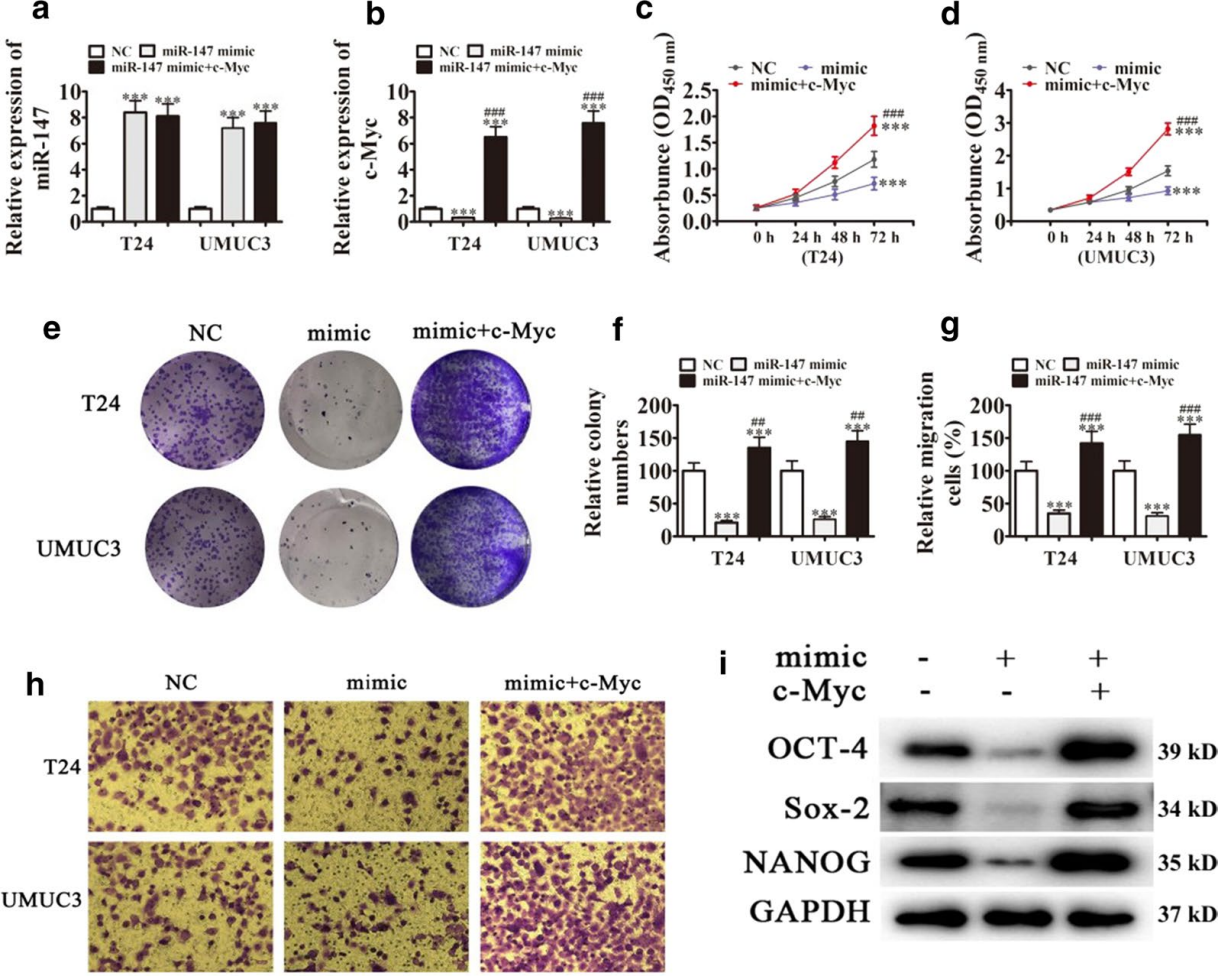
c-Myc overexpression reverses the inhibitory effect of miR-147 on cancer stem cell-mediated BCa cell proliferation and migration in vitro. **a**, **b** qRT-PCR assay showing miR-147 (**a**) and c-Myc (**b**) expression in T24 and UMUC3 cells. Data are presented as the mean ± SD. ***P < 0.001 vs. NC. ^###^P < 0.001 vs. si-circRNA. **c**, **d** CCK8 assays were performed to assess cell proliferation in T24 (**c**) and UMUC3 (**d**) cells. Data are presented as the mean ± SD. *P < 0.05, ***P < 0.001 vs. NC. ^###^P < 0.001 vs. si-circRNA. **e**, **f** Clone formation assay showing the cell proliferation of T24 and UMUC3 cells. Data are presented as the mean ± SD. ***P < 0.001 vs. NC. ^##^P < 0.01, ^###^P < 0.001 vs. si-circRNA. **g**, **h** Cell migration was determined in T24 and UMUC3 cells using Transwell^®^ assays. Data are presented as the mean ± SD. ***P < 0.001 vs. NC. ^###^P < 0.001 vs. si-circRNA. **i** Western blot analysis showing the expression of cancer stem cell-related proteins OCT-4, Sox-2, and NANOG in T24 and UMUC3 cells

We further validated the relationship between c-Myc and miR-147. We constructed miR-147 overexpressing cells with or without c-Myc overexpression. The results showed that miR-147 expression was increased in UMUC-3 and T24 cells transfected with the miR-147 mimic. However, c-Myc overexpression had no effect on miR-147 expression (Fig. 4a). miR-147 overexpression downregulated c-Myc in T24 and UMUC-3 cells transfected with miR-147 mimic. c-Myc overexpression significantly upregulated c-Myc because the heterogeneous transfection of c-My had no 3′-UTR and cannot interact with and be degraded by miR-147 (Fig. 4b).

CCK8 detection (Fig. 4c, d) and colony formation assays (Fig. 4e, f) showed that miR-147 overexpression decreased UMUC-3 and T24 cell proliferation, whereas c-Myc overexpression recovered and increased cell proliferation after miR-147 overexpression. Transwell assays showed that miR-147 overexpression decreased the migration ability of T24 and UMUC-3 cells. However, c-Myc overexpression promoted the migration of T24 and UMUC-3 cells, even after miR-147 overexpression (Fig. 4g, h). Western blot assays showed that miR-147 overexpression downregulated the CSC-related proteins, Sox-2, OCT-4, and NANOG, whereas c-Myc overexpression upregulated CSC-related proteins in T24 and UMUC-3 cells, even after miR-147 overexpression (Fig. 4i).

### Downregulation hsa_circ_0068307 suppresses tumor growth in nude mouse xenografts

**Fig. 5:**
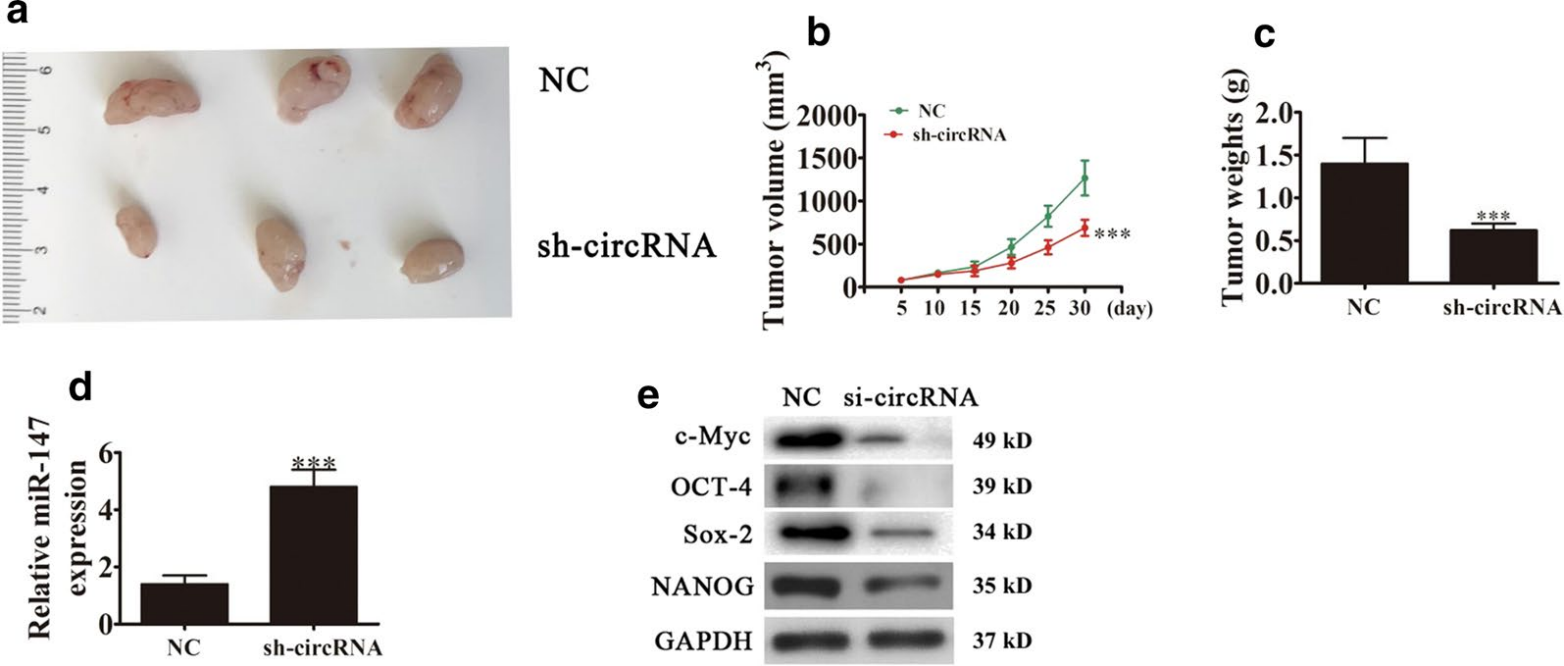
Downregulation hsa_circ_0068307 suppresses tumor growth in nude mouse xenografts. **a** Representative photographs of T24 tumor formation in xenografts of nude mice. n = 6 per group. **b** Summary of tumor volumes in mice measured every week. Data are presented as the mean ± SD. **P < 0.01, ***P < 0.001 vs. NC. **c** Tumor weight was measured after 30 days of injection. Data are presented as the mean ± SD. ***P < 0.001 vs. NC. **d** qRT-PCR assay showing the expression of miR-147. Data are presented as the mean ± SD. ***P < 0.001 vs. control. **e** Western blot analysis of the expression of c-Myc and cancer stem cell-related proteins OCT-4, Sox-2, and NANOG in tumor tissues. Data are presented as the mean ± SD. ***P < 0.001 vs. NC

Finally, we validated the role of hsa_circ_0068307 involved in BCa in vivo. Hsa_circ_0068307 knockdown stable lentiviral strain (small hairpin RNA overexpression vector, sh-circRNA) or sh-NC T24 cells were used for tumor formation. The xenograft results confirmed that hsa_circ_0068307 knockdown suppressed tumor growth, as determined by tumor weight and volume, compared with the NC group (Fig. 5a–c). Rt-qPCR detection showed that hsa_circ_0068307 knockdown upregulated miR-147 expression in tumor tissues (Fig. 5d). Western blot detection showed that hsa_circ_0068307 knockdown downregulated c-Myc and the CSC-related proteins, NANOG, OCT-4, and Sox-2 in tumor tissues (Fig. 5e), which further validated the regulatory role of hsa_circ_0068307/miR-147/c-Myc in in vivo BCa xenograft model.

## Discussion

Increasing evidence indicates that noncoding RNAs (such as circRNAs, miRNAs and lncRNAs) are aberrantly expressed in various cancers, and studies have focused on the role of epigenetic regulation in cancer development [19, 20]. The results of the present study suggested that hsa_circ_0068307 expression is increased in both Bca tissues and cell lines. Downregulation of hsa_circ_0068307 suppressed cell proliferation and migration in both T24 and UMUC-3 cells. These results indicate that hsa_circ_0068307 is involved in BCa development and pathogenesis. To reveal the regulatory mechanism underlying the involvement of hsa_circ_0068307, we predicted that miR-147 and c-Myc were downstream targets of hsa_circ_0068307 using bioinformatics analysis. Luciferase reporter assays confirmed that hsa_circ_0068307 regulated c-Myc expression by sponging miR-147.

miR-147 can suppresses human hepatocellular carcinoma migration, chemosensitivity, and proliferation by inhibiting HOXC6 [21]. miR-147 suppresses migration, proliferation, and invasion in breast cancer via the AKT/mTOR signaling pathway [22]. In the present study, we showed that hsa_circ_0068307 silencing abolished the inhibitory effect of hsa_circ_0068307 on miR-147. Further study suggested that c-Myc was the target of miR-147.

Overexpression of c-Myc promotes the development of cancer [23, 24]. c-Myc contributes to maintain stemness, self-renewal and chemoresistant properties [25, 26]. The present results indicated that downregulation of c-Myc suppressed CSC differentiation in BCa. Moreover, overexpression c-Myc increased the levels of stem cell markers, such as OCT-4, NANOG, and Sox-2. The positive regulation of self-renewal and the maintenance of stemness did not occur after silence hsa_circ_0068307, which decreased c-Myc level by promotion miR-147 expression. In conclusion, hsa_circ_0068307 promoted tumor growth in vitro and in vivo, primarily via sponging miR-147 and promotion c-Myc expression.

## Conclusions

In conclusion, we demonstrated that hsa_circ_0068307 silencing suppressed the transcriptional activation of c-Myc by upregulating miR-147, which downregulated the CSC-related proteins, OCT-4, NANOG, and Sox-2 and led to decreased cell proliferation and migration. We provided several novel strategies for c-Myc inhibition. Additional studies would provide insight into the involvement of the hsa_circ_0068307/miR-147/c-Myc axis in tumorigenesis, which would provide novel therapeutic agents for the treatment of BCa.

## Supplementary information

**Additional file 1.** Images of tumor sphere formation assays in T24 cells (200 cells/well), scale bar, 100 μm.

## Data Availability

The datasets used and/or analyzed during the current study are available from the corresponding author on reasonable request.

## Abbreviations

circRNAs: Circular RNAs
BCa: Bladder cancer
CSCs: Cancer stem cells
FBS: Fetal bovine serum
CCK-8: Cell Counting Kit-8
RT-qPCR: Quantitative reverse transcription-polymerase chain reaction
