# Supplementary figures and images for: Hsa_circ_0068307 mediates bladder cancer stem cell-like properties via miR-147/c-Myc axis regulation
Source: Cancer Cell Int. 2020 May 6;20:151. doi: 10.1186/s12935-020-01235-6 (PMC7204228; doi:10.1186/s12935-020-01235-6)

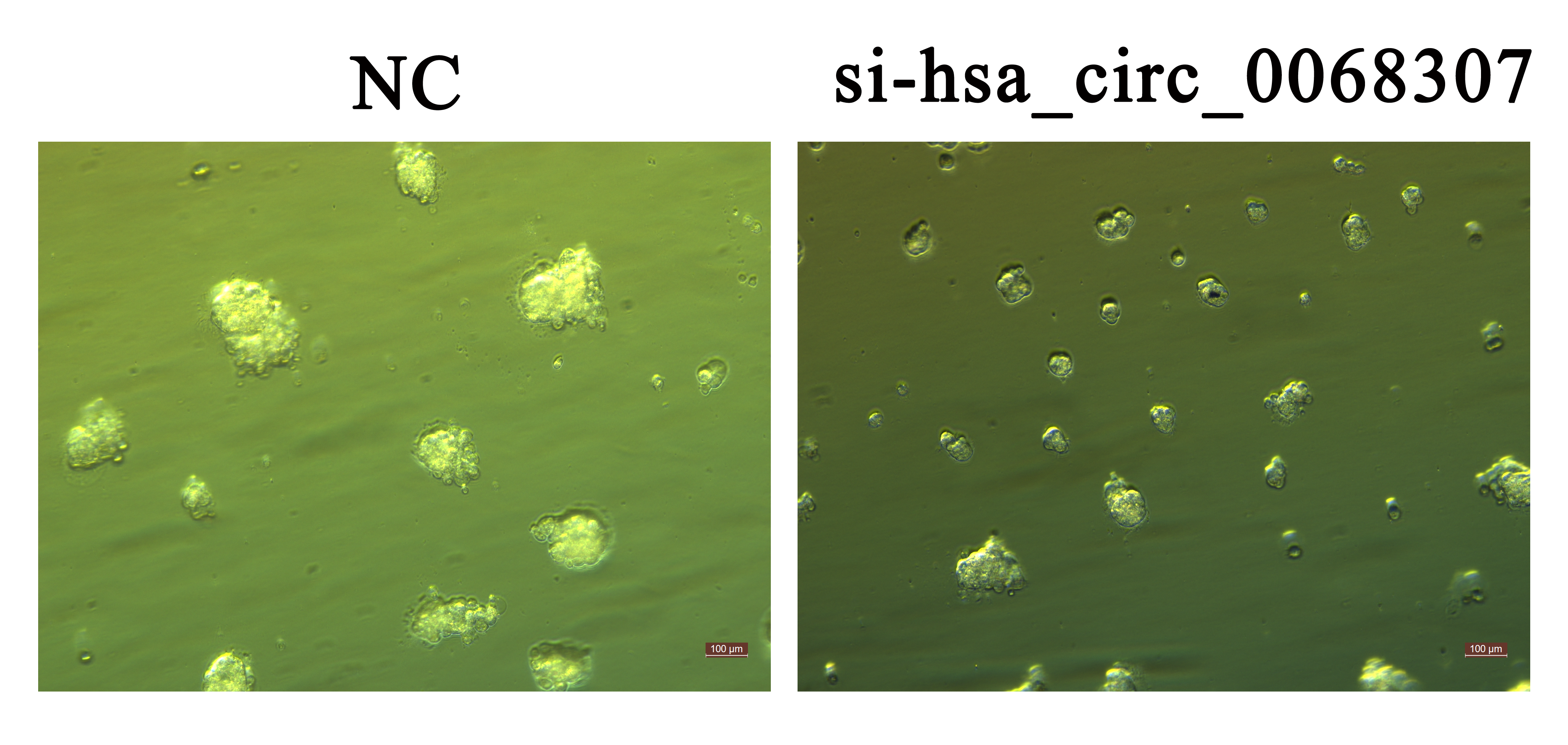

Supplement: Supplementary file 1 — Additional file 1. Images of tumor sphere formation assays in T24 cells (200 cells/well), scale bar, 100 μm. [file 12935_2020_1235_MOESM1_ESM.tif]
